# Protection against Oxygen-Glucose Deprivation/Reperfusion Injury in Cortical Neurons by Combining Omega-3 Polyunsaturated Acid with *Lyciumbarbarum* Polysaccharide

**DOI:** 10.3390/nu8010041

**Published:** 2016-01-13

**Authors:** Zhe Shi, Di Wu, Jian-Ping Yao, Xiaoli Yao, Zhijian Huang, Peng Li, Jian-Bo Wan, Chengwei He, Huanxing Su

**Affiliations:** 1 State Key Laboratory of Quality Research in Chinese Medicine, Institute of Chinese Medical Sciences, University of Macau, Macao 999078, China; zhe.shield@gmail.com (Z.S.); dierwu@gmail.com (D.W.); hzj609@163.com (Z.H.); pengli@umac.mo (P.L.); jianbowan@umac.mo (J.-B.W.); 2 Department of Cardiac Surgery II, The First Affiliated Hospital of Sun Yat-Sen University, Guangzhou 510080, China; jianpingyao@163.com; 3 Department of Neurology, National Key Clinical Department and Key Discipline of Neurology, The First Affiliated Hospital of Sun Yat-Sen University, Guangzhou 510080, China; liliyao71@163.com

**Keywords:** Ca^2+^, cortical neurons, DHA, LBP, OGD/R, neuroprotection, Trk-B

## Abstract

Ischemic stroke, characterized by the disturbance of the blood supply to the brain, is a severe worldwide health threat with high mortality and morbidity. However, there is no effective pharmacotherapy for ischemic injury. Currently, combined treatment is highly recommended for this devastating injury. In the present study, we investigated neuroprotective effects of the combination of omega-3 polyunsaturated fatty acids (ω-3 PUFAs) and *Lyciumbarbarum* polysaccharide (LBP) on cortical neurons using an *in vitro* ischemic model. Our study demonstrated that treatment with docosahexaenoic acid (DHA), a major component of the ω-3 PUFAs family, significantly inhibited the increase of intracellular Ca^2+^ in cultured wild type (WT) cortical neurons subjected to oxygen-glucose deprivation/reperfusion (OGD/R) injury and promoted their survival compared with the vehicle-treated control. The protective effects were further confirmed in cultured neurons with high endogenous ω-3 PUFAs that were isolated from *fat-1* mice, in that a higher survival rate was found in *fat-1* neurons compared with wild-type neurons after OGD/R injury. Our study also found that treatment with LBP (50 mg/L) activated Trk-B signaling in cortical neurons and significantly attenuated OGD/R-induced cell apoptosis compared with the control. Notably, both combining LBP treatment with ω-3 PUFAs administration to WT neurons and adding LBP to *fat-1* neurons showed enhanced effects on protecting cortical neurons against OGD/R injury via concurrently regulating the intracellular calcium overload and neurotrophic pathway. The results of the study suggest that ω-3 PUFAs and LBP are promising candidates for combined pharmacotherapy for ischemic stroke.

## 1. Introduction

Ischemic stroke, characterized by the disturbance of the blood supply to the brain, is a severe worldwide health threat with high mortality and morbidity [1]. However, there is no safe and effective pharmacotherapy for ischemic injury. At present, neuroprotection remains the central focus of ischemic stroke treatment after reperfusion [2]. Despite considerable research effort, the development of a suitable neuroprotective agent to treat ischemic stroke usually failed when transitioned to the clinical utilization [3]. Therefore, combined treatment is highly recommended for this devastating injury [4].

Omega-3 polyunsaturated fatty acids (ω-3 PUFAs) have been demonstrated to elicit therapeutic effects in a variety of neurological disorders including ischemic stroke [5,6,7,8,9,10]. They are essential fatty acids for human beings, which can maintain cellular membrane structural and functional integrity. Several lines of evidence have suggested that the anti-inflammation and anti-apoptosis action may account for the neuroprotective effects of ω-3 PUFAs [11,12,13,14,15]. It is evident that mammals cannot synthesize ω-3 PUFAs due to the lack of a fatty acid desaturase [16]. Kang *et al.* engineered a transgenic mouse carrying a *fat-1* gene from *Caenorhabditiselegans* [17], which encodes the enzyme to convert ω-6 into ω-3 PUFAs and enable the animal to maintain a steady ω-3 PUFAs level. Thus, the use of the *fat-1* transgenic mouse provides a unique chance to study the beneficial effects of endogenous ω-3 PUFAs. Moreover, abundant studies have reported that *Lyciumbarbarum* polysaccharide (LBP), a major active ingredient of *Lyciumbarbarum*, has anti-apoptotic effects in resisting ischemic cerebral injury both *in vitro* and *in* *vivo* [18,19]. Although the anti-apoptotic effects of LBP have been extensively demonstrated [18,20,21], no clear evidence has been provided to illustrate how LBP triggers the intracellular anti-apoptotic signal cascade. Therefore, we infer that LBP may exert its neuroprotection through a unique way different from ω-3 PUFAs. Thus, the combined therapies with ω-3 PUFAs and LBP could display a better curative effect in ischemia treatment.

Oxygen-glucose deprivation/reperfusion (OGD/R) is an *in vitro* model that mimics the *in vivo* ischemia/reperfusion injury. The reperfusion after transient deprivation of oxygen and glucose disrupts the permeability of cell membrane and eventually leads to neuronal cell death. Various interventions have been used to protect cells after OGD/R injury such as maintaining intracellular Ca^2+^ level and activating Trk receptor tyrosine kinases [22,23], since Ca^2+^ overloading is a main event which results into increased cell vulnerability and oxidative stress in the progress of apoptosis and Trk receptor tyrosine kinases, a family of transmembrane-receptor signaling systems, can subsequently trigger downstream signal pathways to induce pro-survival effects.

In the present study, we investigated the neuroprotective effects of ω-3 PUFAs, LBP and the combination of ω-3 PUFAs and LBP on rescuing cortical neurons from OGD/R and determined their distinguishing mechanisms of action through particularly activating Trk B receptor and reducing intracellular Ca^2+^ overload.

## 2. Materials and Method

### 2.1. Animals

Experimental mice were obtained by mating male *fat-1* mice (C57BL/6 background obtained from Dr. Jing X. Kang, Harvard Medical School, MA, USA) and female C57BL/6 wild type (WT) mice. Mice were fed a modified diet containing 10% corn oil (TROPHIC Animal Feed High-tech Co., Ltd, Nantong, China), with a fatty acid profile rich in ω-6 (mainly linoleic acid) and low in ω-3 PUFAs (~0.1% of the total fat supplied). Food and water were given freely until the desired age for primary neuron cultures (E16-18). All animal experiments were carried out in strict accordance with the ethical guidelines of Institute of Chinese Medical Science (ICMS), University of Macau.

### 2.2. Primary Cortical Neuron Cultures and Oxygen-Glucose Deprivation/Reperfusion (OGD/R)

Cortical cultures were obtained from E16.5 WT or *fat-1* embryos. The presence of the *fat-1* gene was confirmed by genotyping on each embryo. Cerebral cortices were removed, and stripped of meninges. Tissues were digested in 0.05% trypsin, and triturated. Cells were seeded in 6- or 24-well plates pre-treated with poly-l-lysine and laminin (Sigma-Aldrich, Saint Louis, MS, USA). Cultures were maintained in Neurobasal medium containing 2% B27 supplement and 0.5 mM GlutaMAX™-I (Life Technologies, Carlsbad, CA, USA). Cultures were kept at 37 °C, 100% humidity and in a 95% air/5% CO_2_ atmosphere. Unless indicated, experiments were performed after 7 days *in vitro* (DIV 7).

For OGD/R, cultures were placed in a hypoxia chamber containing an atmosphere of <0.2% O_2_, 5% CO_2_, 95% N_2_, >90% humidity, and 37 °C. Within the chamber, the medium was removed and replaced with oxygen/glucose-free balanced salt solution (BSS, in mmol/L: 116 mM NaCl, 5.4 mM KCl, 0.8 mM MgSO_4_, 1 mM NaH_2_PO_4_·2H_2_O, 262 mM NaHCO_3_, 1.8 mM CaCl_2_, pH 7.2, <0.1% O_2_), which was previously saturated with 95% N_2_/5% CO_2_ at 37 °C. Still within the chamber, cells were washed twice with oxygen/glucose-free BSS. Cultures were taken out of the chamber after 4 h and transferred to the regular cell culture incubator. Sham-treated cultures were always handled in parallel and received similar wash steps as OGD/R-treated cultures with the difference in that BSS contains 4.5 g/L glucose and regular oxygen.

### 2.3. Drugs

The preparation for LBP extracts was the same as reported previously [24]. LBP (50 mg/L) was dissolved into primary neuron culture medium immediately before use.

DHA was dissolved into 100% ethanol and stored at −20 °C in the dark as described in previous study [25]. A concentration of 10 μM was selected based on our previous finding [26]. Immediately before use, the DHA stock solution was diluted in the bath solution and adjusted to the final concentrations needed.

### 2.4. Antibodies

Rabbit anti-GFAP monoclonal antibody and mouse anti-β-tubulin III monoclonal antibody were supplied by Sigma-Aldrich (Sigma-Aldrich). Goat anti-mouse 488 and goat anti-rabbit 568 secondary antibody were obtained from Life Technologies.

Primary antibodies of goat anti-Trk-B, rabbit anti-Bcl-2 andrabbit anti-GADPH were purchased from Cell Signaling Technology (Cell Signaling Technology, Boston, MD, USA). Horseradish peroxidase secondary antibodies were from Beyotime (Beyotime, Jiangsu, China).

### 2.5. Immunocytochemistry

Cell types were characterized by immunocytochemistry. Tuj-1 was used as marker for neurons while GFAP for astrocytes. Briefly, neurons were fixed by 4% paraformaldehyde, blocked with 10% goat serum. Primary antibodies of Tuj-1 (1:500) and GFAP (1:500) diluted in blocking buffer were incubated with cells at 4 °C overnight. After PBS washing, appropriate secondary antibodies were added at room temperature in the dark, followed with DAPI counterstaining. Immunostaining was analyzed using a fluorescence microscope (Leica DM6000 B) interfaced with a digital camera and an image analysis system.

### 2.6. Genomic DNA Extractions and PCR Amplification

The *fat-1* phenotypes of each animal were characterized using isolated genomic DNA. Genomic DNA was prepared from collections of embryo brain tissues using DNA Isolation Kits. The DNA was used running polymerase chain reactions (PCR) using oligonucleotide primers that are specific for the transgene. Primer pair sets for the fat-1 gene were constructed from Invitrogen (Genewiz, Beijing, China) as follows: *Fat-1* forward: 5′-TGTTCATGCCTTCTTCTTTTTCC-3′; reverse: 5′-GCGACCATACCTCAAACTTGGA-3′. PCR was carried out using rTaq with the following conditions: 95 °C 60 s (1 cycle); 95 °C 20 s, 58 °C 30 s, 72 °C 40 s (34 cycles). Amplified fragments were separated by 1.5% agarose gel electrophoresis.

### 2.7. Fatty Acid Analysis

To examine whether the expression of the *fat-1* gene altered the PUFA composition in the primary cultured cortical neurons of the *fat-1* and WT groups, fatty acid analysis were processed by using gas chromatography-mass spectrometry (GC-MS), as described previously [27]. Briefly, cell samples were ground to powder under liquid nitrogen and subjected to fatty acid methylation by 14% boron trifluoride-methanol reagent at 100 °C for 1 h. Fatty acid methyl esters were analyzed by an Agilent GC-MS system (Agilent Technologies, Palo Alto, CA, USA) consisting of an Agilent 6890 gas chromatography and an Agilent 5973 mass spectrometer. Fatty acids were identified in forms of their methyl esters by three means: (i) searching potential compounds from NIST MS Search 2.0 database; (ii) comparing retention time with those of reference compounds (Nu-Chek Prep, Elysian, MN, USA) eluted under the identical chromatographic condition; and (iii) comparing their mass spectra plots with those of authentic standards. Quantification was performed by normalizing individual peak area as the percentage of total fatty acids.

### 2.8. Cell Viability Assay

Cell viability was assessed using a Cell Counting Kit-8 (CCK-8) dye (Dojindo Laboratories, Japan) according to the manufacturer’s instructions. Briefly, after 10 μL of CCK-8 solution was add to each well, cells were incubated at 37 °C for 30 min and the absorbance was finally determined at 450 nm using a microplate reader. The results were expressed as relative cell viability (%).

### 2.9. TUNEL Staining

To identify apoptotic neurons, TUNEL assays were performed using an *in situ* cell death detection kit (Roche, No. 11 684 795 9101). After washed three times by ice-cold PBS, the cell samples were fixed with a freshly prepared fixation solution for 1h and incubated in permeabilization solution for 2 min on ice. Then, 50 μL TUNEL reaction mixture was added on each sample. Slides were incubated in a humidified atmosphere for 60 min at 37 °C in the dark, followed by counterstaining with DAPI. The number of TUNEL-positive cells was counted in 10 randomized fields per well under a fluorescence microscope. Results were the average ± SEM of data from 5 experiments unless stated otherwise in the legends.

### 2.10. Intracellular Calcium (Ca^2+^) Measurements

Intracellular Ca^2+^ imaging was conducted using a Fluo4-AM dye (Dojindo Laboratories), which has strong ability to combine with free calcium ions inside living cells.

After washing 3 times with HBSS, cells prepared in 96-well plates were incubated with Fluo 4-AM working solution at 37 °C for 60 min. Washed 3 times to clean up the remains of Fluo 4-AM, cells were covered by HBSS for another 30 min at 37 °C to make deesterification of AM completely. At last, cells were analyzed under a fluorescence microscope (Leica DM6000 B) interfaced with a digital camera and an image analysis system. Images were taken under same aperture and speed. Ten pictures of each group were randomly selected and software Image Pro plus 6.0 was used to measure the intensity of each photo.

### 2.11. Western Blotting Analysis

Cortical neurons in 6cm dishes were washed with ice-cold PBS for 3 times and lysed with a lysis buffer containing protease inhibitors (Beyotime, Jiangsu, China) at 24 h after OGD/R treatments. The protein concentration was determined using a BCA protein assay kit. Then, protein extracts were separated by electrophoresis on 12% SEMS-polyacrylamide gel electrophoresis (SEMS-PAGE) gels and transferred onto polyvinylidene fluoride (PVDF) membranes. The membranes were sequentially incubated with primary antibodies and secondary antibodies, and enhanced chemiluminescence (ECL) solution and followed by autoradiography. The intensity of the blots was analyzed using Image Pro plus 6.0.

### 2.12. Statistical Analysis

The results were expressed as the mean ± SEM of triplicate measurements representative of three independent experiments. Multiple group comparisons were made by one-way ANOVA followed with Tukey *post hoc* test. Statistical significance was defined as *p* < 0.05.

## 3. Results

### 3.1. Identification, Genotyping and Fatty Acid Profiles of Primary Cortical Neurons

**Figure 1:**
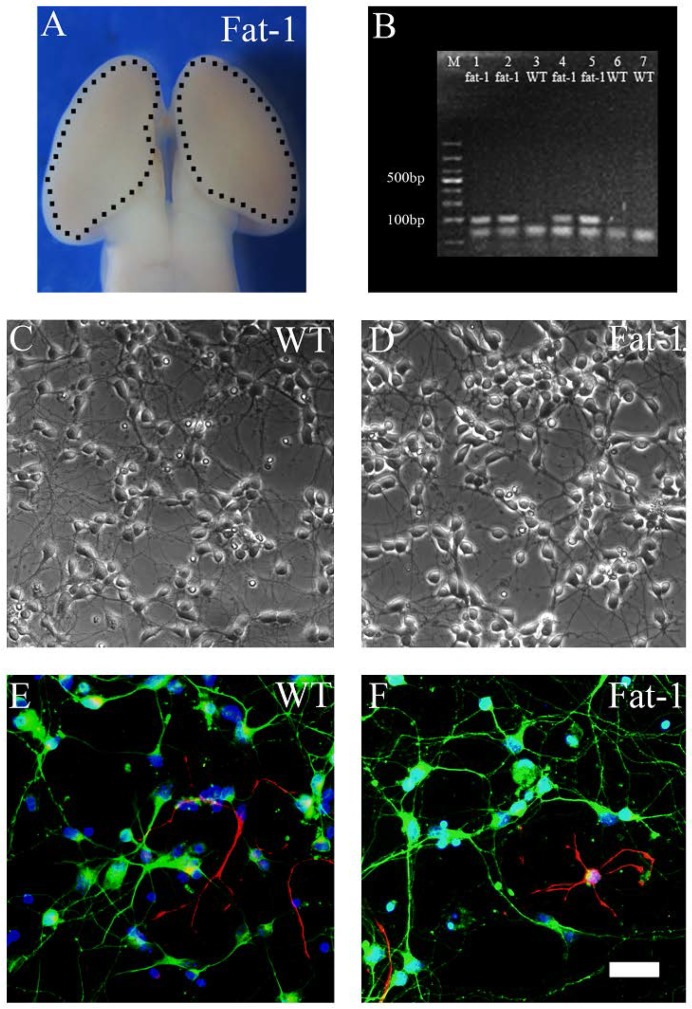
Identification of primary cultured neurons. Cultures were prepared from the cortex of E16.5 *fat-1* and WT embryos and examined at 7 DIV. (**A**) An image showing the cortical tissue in the embryonic brain; (**B**) gel electrophoresis of PCR products using primers for *fat-1* gene. Wild-type controls (lanes 3, 6 and 7) and positive fat-1 specimens (lanes 1, 2, 4 and 5); (**C**,**D**) examples of phase contrast images of cultured primary neurons; and (**E**,**F**) images showing immunostainning on WT and *fat-1* neurons respectively (**Green**, β-III tubulin; **Red**, GFAP; **Blue**, DAPI). Scale bar: 50 µm.

Primary cortical neurons were derived from E16.5 mice embryos (Figure 1A). Figure 1B shows the genotyping results of each embryo tissues. PCR analysis demonstrated the high expression of *fat-1* gene (lanes 1, 2, 4 and 5) in *fat-1* embryo tissues while no expression was found in WT embryo tissues (lanes 3, 6 and 7). As shown in Figure 1C,D, the neurons showed distinct cell bodies with synaptic connections. No obvious morphologic difference was observed between WT and *fat-1* derived neurons. Before OGD/R, immunocytochemistry was conducted using β-III tubulin and GFAP antibodies. The majority of cells were β-III tubulin-positive (>95%) and only a very small proportion were GFAP-positive in both *fat-1* neurons (Figure 1E) and WT neurons (Figure 1F). Fatty acid analyses of cultured primary neurons were performed using GC-MS. As shown in Table 1, *fat-1* neurons exhibited increased expression of ω-3 PUFAs including DPA and DHA (** *p* < 0.01 compared with WT neurons) with a significant decrease in overall ω-6/ω-3 PUFA ratio compared with WT neurons.

**Table 1:** Profiles of polyunsaturated fatty acid of primary cortical neurons derived from *fat-1* transgenic embryos and their WT littermates.

Fatty Acid	WT	*fat-1*
C14:0	3.01 ± 0.25	1.77 ± 0.33 *
C16:0	25.22 ± 0.27	24.20 ± 0.46
C16:1,9	8.65 ± 0.23	5.32 ± 0.57 **
C18:0	13.12 ± 0.85	16.11 ± 0.21 *
C18:1,9	33.12 ± 0.34	28.97 ± 0.12 **
C18:2,6	0.78 ± 0.23	0.77 ± 0.02
C18:3,3 (ALA)	0.11 ± 0.06	0.47 ± 0.11 **
C20:0	0.30 ± 0.05	0.27 ± 0.02
C20:1,9	1.22 ± 0.03	1.14 ± 0.02
C20:2,6	4.661 ± 0.24	2.88 ± 0.41 *
C20:4,6 (AA)	5.11 ± 0.18	0.88 ± 0.09 **
C20:5,3 (EPA)	0.33 ± 0.00	3.79 ± 0.73 **
C22:0	0.20 ± 0.03	0.54 ± 0.09 *
C22:1,9	3.56 ± 0.70	3.95 ± 0.63
C22:5,3 (DPA)	0.99 ± 0.03	4.37 ± 0.31 **
C22:6,3 (DHA)	1.02 ± 0.14	2.90 ± 0.03 **
C24:1	0.92 ± 0.12	1.32 ± 0.17
SFA	41.85 ± 0.34	42.89 ± 1.09
MUFA	47.47 ± 1.25	40.70 ± 1.09 **
PUFA	13.00 ± 1.05	16.06 ± 1.72 *
ω-6/ω-3	4.31 ± 4.03	0.39 ± 0.26 **

Data expressed as mol % of total fatty acids ± SEM (* *p* < 0.05 compared with WT; ** *p* < 0.01 compared with WT). Abbreviations: AA, arachidonic acid; ALA, alpha linolenic acid; DHA, docosahexaenoic acid; DPA, docosapentaenoic acid; EPA, eicosapentaenoic acid; LA, linoleic acid; MUFA, monounsaturated fatty acids (the value is given as follows: C16:1 + C18:1 + C20:1 + C22:1 + C24:1); SFA, saturated fatty acids (the value is given as follows: C14:0 + C16:0 + C18:0 + C20:0 + C22:0); PUFA, polyunsaturated fatty acids.

### 3.2. LBP Either Together with DHA or Endogenous ω-3 PUFAs Rescues Cortical Neurons from OGD/R Insults

**Figure 2:**
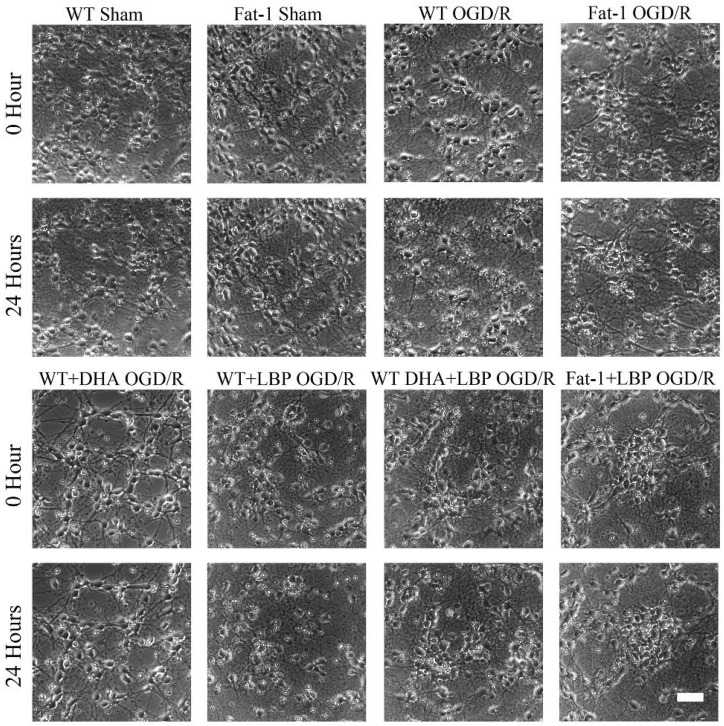
Primary cortical neurons were protected against OGD/R injury after LBP and ω-3 PUFAs treatment. Phase contrast images showing the morphological changes of the primary cultured neurons prior or post OGD/R injury. Scale bar: 50 µm.

To examine whether the combination of ω-3 PUFAs and LBP can promote neuronal survival under ischemia/reperfusion conditions, we induced OGD/R injury on cultured neurons at 7 DIV. The cultured neurons were exposed to a hypoxic and glucose-free environment for 3 h, followed by normal culture for 24 h to mimic ischemia/reperfusion injury. As shown in Figure 2, cortical neurons exhibited typical cell shrinkage and neurite blebbing, and a marked decrease in the cell number at 24 h after OGD/R injury. The bright hollows on the phase contrast images indicated an injury status of neurons after reperfusion, in which the most severe situation goes to WT OGD/R group. Conversely, neurons in all treatment groups showed intact cell bodies with elaborate networks of neuritis and remarkably attenuated OGD/R-induced morphological abnormalities compared with WT OGD/R neurons.

### 3.3. LBP Either Together with DHA or Endogenous ω-3 PUFAs Significantly Prevents OGD/R-Induced Neuronal Apoptosis via Intracellular Ca^2+^ Handling or Neurotrophic Pathway Activation

**Figure 3:**
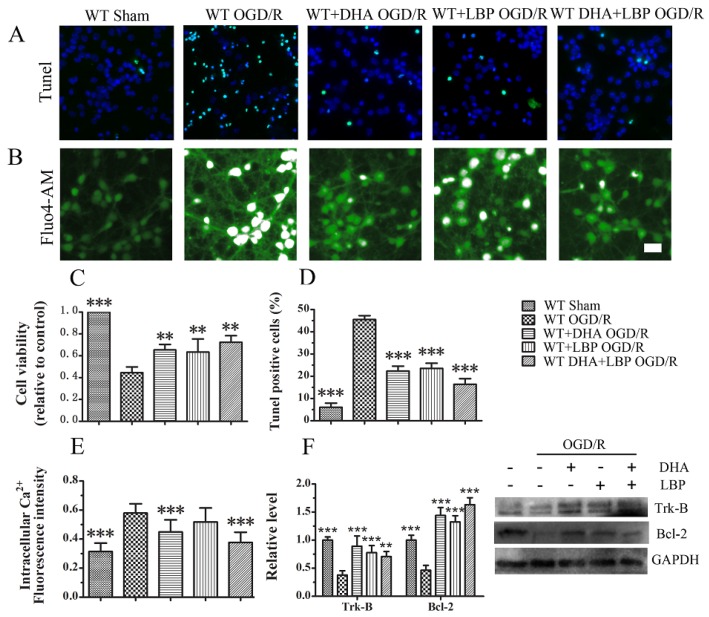
LBP and exogenous DHA (10 μM) significantly prevent OGD/R-induced neuronal apoptosis respectively via intracellular Ca^2+^ handling or neurotrophic pathway activation: (**A**) TUNEL staining; (**B**) fluorescent micrographs showing intracellular Ca^2+^ levels as stained by the Fluo4-AM dye; (**C**) statistic of cell viability; (**D**) statistic of TUNEL positive cells; (**E**) results of relative fluorescence intensity analysis of intracellular Ca^2+^; and (**F**) expression levels of Trk-B and Bcl-2 measured by Western blot. Data are presented as mean ± SEM, ** *p* < 0.01, *** *p* < 0.001 indicate significant difference compared with the WT OGD group; *p* < 0.05 indicates significant difference compared with the WT DHA + LBP group (*t*-test). Scale bar: 50 µm.

The neuroprotective effects of combination of exogenous DHA and LBP were determined first. Cell viability was determined using a CCK-8 assay. OGD/R insults resulted in severe cell death in WT model group (approximately 45%). All the single treatment groups displayed a significant reduced neuronal death after OGD/R insults in that neuronal death was reduced to 34.7% in DHA-treated group and 36.6% in LBP-treated group. Notably, LBP combined with DHA further reduced neuronal cell death to 27.6% (Figure 3C). As shown in Figs. 3A and D, OGD/R induced approximately 50% TUNEL-positive cells in WT neurons. Cells in green fluorescence indicated TUNEL-positive and represented the apoptotic cells. Nuclei were labeled in blue with DAPI stands for the total number of cells in the present vision field. The ratio of apoptotic neurons was remarkably decreased in the culture of LBP- and DHA-treated WT neurons in which less TUNEL-positive cells were found after OGD/R injury (23.6% in LBP-treated group and 22.4% in DHA-treated group). Interestingly, LBP combined with DHA further reduced apoptosis after OGD/R insults (16.4%), indicating that a combined treatment exerts the maximal effect on protecting neurons against OGD/R injury among all the treatment groups. Moreover, Ca^2+^ ion plays an important role in maintaining the normal function of neurons. The concentration of Calcium ion remains a significant difference between the cell membranes and while injured, will be elevated from extracellular environment or the release of mitochondrion. Therefore, a constant rise in intracellular Ca^2+^ reflects the impaired situation of cells. Figure 3B illustrated the effect of different treatment on intracellular Ca^2+^ concentration. The results showed that WT OGD/R group displayed significant higher fluorescence intensity. Although the concentration of Ca^2+^ was slightly lower in single LBP treated group compared with WT OGD/R group, no significant statistics difference was observed between these two groups under the present experimental conditions. Furthermore, consistent with previous reports, the Fluo-4 fluorescence intensity was decreased by exogenous DHA treatment compared with WT OGD/R neurons. Intriguingly, our data demonstrated that combined use of LBP with exogenous DHA could further reduce Ca^2+^ levels, which implied a better effect on preventing Ca^2+^ overloading even compared with either single DHA treated group. Then, the expression levels of Trk-B receptor as well as Bcl-2 were determined by western blot assay. As shown in Figure 3F, the expression of Trk-B receptor and Bcl-2 were significantly decreased in WT OGD/R group. Both LBP and DHA treatment could remarkably reverse the reduction of Trk-B and Bcl-2 expression. Our data indicated that LBP might possibly exert its neuroprotection by activating Trk-B receptor and consequently initiate the pro-survival cascade. In addition, combined use of LBP together with exogenous DHA displayed an enhanced effect on activating Trk-B expression.

**Figure 4:**
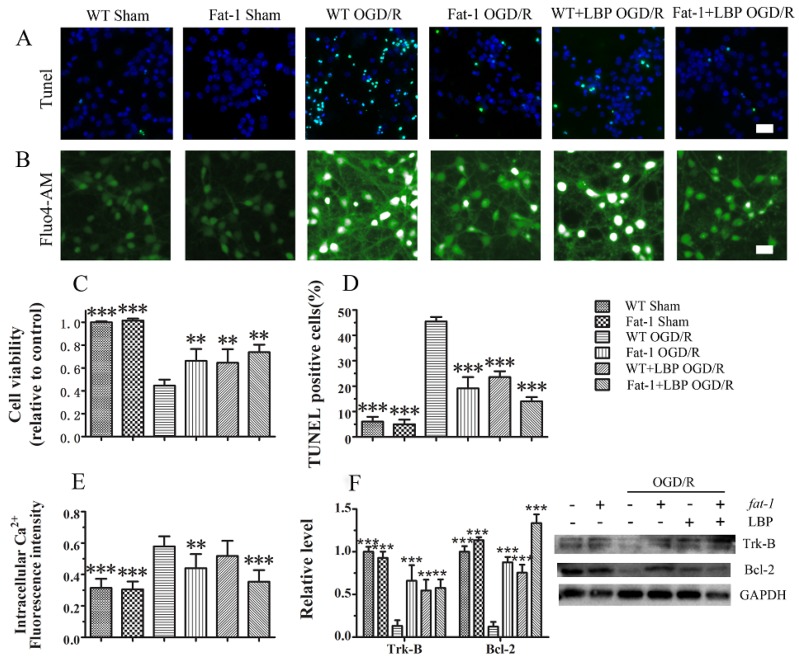
LBP and endogenous ω-3 PUFAs significantly prevent OGD/R-induced neuronal apoptosis respectively via intracellular Ca^2+^ handling or neurotrophic pathway activation: (**A**) TUNEL staining; (**B**) fluorescent micrographs showing intracellular Ca^2+^ levels as stained by the Fluo4-AM dye; (**C**) statistic of cell viability; (**D**) statistic of TUNEL positive cells; (**E**) results of relative fluorescence intensity analysis of intracellular Ca^2+^; and (**F**) expression levels of Trk-B and Bcl-2 measured by Western blot. Data are presented as mean ± SEM, ** *p* < 0.01, *** *p* < 0.001 indicate significant difference compared with the WT OGD group; *p* < 0.05, *p* < 0.01 indicates significant difference compared with the WT DHA + LBP group (*t*-test). Scale bar: 50 µm.

Afterward, the protective effects were further confirmed in cultured neurons with high endogenous ω-3 PUFAs, which were isolated from *fat-1* mice, in that a higher survival rate was found in *fat-1* neurons compared with wild-type neurons after OGD/R injury. As shown in Figure 4C, all the single treatment groups displayed a significantly reduced neuronal death after OGD/R insults in that neuronal death was reduced to 35.5% in LBP-treated group and 33.9% in *fat-1* group. LBP combined with endogenous ω-3 PUFAs further reduced neuronal cell death to 26.3%. As shown in Figure 4A,D, the ratio of apoptotic neurons was remarkably decreased in the culture of LBP-treated WT neurons in which less TUNEL-positive cells were found after OGD/R injury (approximately 24%). The ration of apoptotic neurons in *fat-1* neurons (19.2%) was significantly decreased compared with WT neurons, suggesting that endogenous ω-3 PUFAs have protective effects against OGD/R injury. Interestingly, LBP combined with endogenous ω-3 PUFAs further reduced apoptosis after OGD/R insults (14.0%), which confirmed the enhanced nruroprotective effects of the combined treatment on protecting neurons against OGD/R injury. Furthermore, the results in Figure 4B,E showed that the Fluo-4 fluorescence intensity was decreased by endogenous ω-3 PUFAs treatment compared with WT OGD/R neurons. Our data demonstrated that combined use of LBP with endogenous ω-3 PUFAs could further reduce Ca^2+^ levels as well. Finally, as shown in Figure 4F, the expression of Trk-B receptor and Bcl-2 were significantly decreased in WT OGD/R group. Both LBP and endogenous ω-3 PUFAs could remarkably reverse the reduction of Trk-B and Bcl-2 expression in treated groups. The results further confirmed that combined use of LBP together with endogenous ω-3 PUFAs could enhance their neroprotective effects via activating Trk-B expression.

## 4. Discussion

In the present study, we firstly determined the neuroprotective effect of docosahexaenoic acid (DHA), a major component of the ω-3 PUFA family, together with LBP in primary cortical neurons against OGD/R insult. The deprivation of oxygen and glucose results in the initiation of the depicted ischemic cascade that eventually leads to neuronal death [28]. Because of the loss of nutrients and oxygen, neurons are injured and a lot of devastating cascades are initiated, such as excessive excitatory amino acid release, generation of reactive oxygen species (ROS), expression of pro-apoptotic factors, mitochondrial dysfunction, as well as inflammation [29]. The OGD/R model provides a chance to dissect cellular events that occur after withdrawal of oxygen and glucose and mimic the key pathophysiological events of ischemia *in vivo*. Consistent with previous findings, both substances as well as the combined treatment significantly rescued cortical neurons from OGD/R insults [19,30].

Alterations in Ca^2+^ homeostasis, including mitochondrial Ca^2+^ overload, lead to increased cell vulnerability and oxidative stress [22]. Excessive Ca^2+^ entry ultimately induces acute or delayed neuronal death [31]. It has been reported that ω-3 PUFAs inhibited endoplasmic reticulum (ER) Ca^2+^ release in astrocyte after *in vitro* ischemia [32] and delayed Ca^2+^-induced mitochondrial permeability transition pore opening in myocardium [33]. These findings suggest that ω-3 PUFAs have a potential to reduce intracellular Ca^2+^ overloading. Although LBP was observed to reduce 6-OHDA -induced elevation of intracellular Ca^2+^ in PC12 cells [20], no clear description on the location where Ca^2+^ accumulated was recorded. In the present research, we observed that DHA significantly inhibited the increase of intracellular Ca^2+^, whereas single LBP treatment had limited influence on intracellular Ca^2+^ handling. The mitochondrial apoptosis pathway is controlled by pro- and anti-apoptotic Bcl-2 family proteins and either overexpression of anti-apoptotic Bcl-2, or gene deficiency in the proapoptotic bax gene to prevent excitotoxic apoptosis [34,35]. It is established that increasing the expression level of Bcl-2 can obviously reduce the impact of stroke in neuroprotective treatments [36,37,38]. Consistently, our observations demonstrated that both LBP and ω-3 PUFAs exert their neuroprotection via activating Bcl-2 anti-apoptotic cascade. Additionally, several lines of evidences have demonstrated that modulating Bcl-2 family proteins can only contribute to maintaining Ca^2+^ homeostasis in the ER [39,40]. It can be inferred that the confined alteration in Ca^2+^ contents has limited contribution to the entirety intracellular Ca^2+^ homeostasis. Therefore, this notion may possibly account for the limited impact of LBP on intracellular Ca^2+^ handling observed in our research.

To further determine how LBP and ω-3 PUFAs trigger intracellular pro-survival signaling, we examined the alterations of Trk-B receptors. The Trk receptor tyrosine kinases is a family of transmembrane-receptor signaling systems which promote the development and survival of neurons [41]. Trk-B receptor can be activated by specifically binding with BDNF. The activated Trk-B receptor subsequently triggers downstream signal pathway to induce pro-survival effects [23]. Enriched dietary ω-3 PUFAs has been reported to increase Trk-B mRNA expression in the cerebral cortex [42]. We noticed that both LBP and DHA treatment significantly increased the expression of Trk-B receptors in primary cultured cortical neurons suffered OGD/R insults. In the present study, we reported for the first time that LBP possibly protected neuron from OGD/R-induced apoptosis via modulating neurotrophin pathway, which initiated from the cell membrane. Notably, combined treatment of DHA and LBP showed the maximal effect on protecting cortical neurons against OGD/R injury via concurrently regulating the intracellular calcium accumulation and neurotrophic pathway.

In addition, the protective effects were further confirmed in neurons with high content of endogenous ω-3 PUFAs that were isolated from *fat-1* mice embryos. Dietary supplementation is a conventional approach to increase tissue content of ω-3 PUFAs in animal studies. However, inconsistent results were occasionally observed either due to the variance in the component of dietary supplement or the neglected relevance of the ω-3/ω-6 PUFAs ratio. Kang *et al.* engineered a transgenic mouse carrying a *fat-1* gene from *Caenorhabditiselegans* [17]. The *fat-1* gene encodes a fatty acid desaturase not normally present in mammals, which can convert ω-6 into ω-3 PUFAs. The highly expression of the *fat-1* gene leads to enrichment in endogenous ω-3 PUFAs levels and concomitantly decreased ω-6 PUFAs levels [43]. The use of *fat-1* mice embryos provides a strictly controlled model to investigate the biological properties of ω-3 PUFAs with stable content [44]. The present findings indicated that endogenous ω-3 PUFAs, combining with LBP treatment, exerted a better neuroprotective effect on OGD/R insulted neurons.

In conclusion, we observed the protective effect of ω-3 PUFAs or LBP on enhancing the survival of cultured cortical neurons using an *in vitro* OGD/R model and further demonstrated that a combined treatment of ω-3 PUFAs and LBP exerted the maximal effect on protecting neurons against OGD/R injury. The results of the study suggest that ω-3 PUFAs and LBP are promising candidates for combined pharmacotherapy for ischemic stroke.
